# STAGER checklist: Standardized testing and assessment guidelines for evaluating generative artificial intelligence reliability

**DOI:** 10.1002/imo2.7

**Published:** 2024-07-02

**Authors:** Jinghong Chen, Lingxuan Zhu, Weiming Mou, Anqi Lin, Dongqiang Zeng, Chang Qi, Zaoqu Liu, Aimin Jiang, Bufu Tang, Wenjie Shi, Ulf D. Kahlert, Jianguo Zhou, Shipeng Guo, Xiaofan Lu, Xu Sun, Trunghieu Ngo, Zhongji Pu, Baolei Jia, Che Ok Jeon, Yongbin He, Haiyang Wu, Shuqin Gu, Wisit Cheungpasitporn, Haojie Huang, Weipu Mao, Shixiang Wang, Xin Chen, Loïc Cabannes, Gerald Sng Gui Ren, Iain S. Whitaker, Stephen Ali, Quan Cheng, Kai Miao, Shuofeng Yuan, Peng Luo

**Affiliations:** ^1^ Department of Oncology, Zhujiang Hospital Southern Medical University Guangzhou China; ^2^ The Second School of Clinical Medicine Southern Medical University Guangzhou China; ^3^ Department of Urology, Shanghai General Hospital Shanghai Jiao Tong University School of Medicine Shanghai China; ^4^ Department of Oncology, Nanfang Hospital Southern Medical University Guangzhou China; ^5^ Institute of Logic and Computation, TU Wien Wien Austria; ^6^ Institute of Basic Medical Sciences Chinese Academy of Medical Sciences and Peking Union Medical College Beijing China; ^7^ Department of Urology, Changhai Hospital Naval Medical University (Second Military Medical University) Shanghai China; ^8^ Department of Radiation Oncology, Zhongshan Hospital Fudan University Shanghai China; ^9^ Molecular and Experimental Surgery, University Clinic for General‐, Visceral‐, Vascular‐ and Trans‐Plantation Surgery, Medical Faculty University Hospital Magdeburg Otto‐von Guericke University Magdeburg Germany; ^10^ Department of Oncology The Second Affiliated Hospital of Zunyi Medical University Zunyi China; ^11^ Translational Radiobiology, Department of Radiation Oncology Universitätsklinikum Erlangen Erlangen Germany; ^12^ Comprehensive Cancer Center Erlangen‐EMN Erlangen Germany; ^13^ GZDLab Chongqing China; ^14^ Department of Cancer and Functional Genomics, Institute of Genetics and Molecular and Cellular Biology CNRS/INSERM/UNISTRA Illkirch France; ^15^ Linguistique Informatique, UFR‐Linguistique Université Paris Cité Paris France; ^16^ Xianghu Laboratory Hangzhou China; ^17^ Department of Life Science Chung‐Ang University Seoul Korea; ^18^ School of Sport Medicine and Rehabilitation Beijing Sport University Beijing China; ^19^ Department of Microbiology and Immunology, School of Medicine, Lineberger Comprehensive Cancer Center University of North Carolina at Chapel Hill Chapel Hill North Carolina USA; ^20^ Department of Graduate School Tianjin Medical University Tianjin China; ^21^ Department of Clinical College of Neurology, Neurosurgery and Neurorehabilitation Tianjin Medical University Tianjin China; ^22^ Duke Human Vaccine Institute Duke University Medical Center Durham North Carolina USA; ^23^ Department of Medicine Mayo Clinic Rochester New York USA; ^24^ Department of Biochemistry and Molecular Biology Mayo Clinic College of Medicine and Science Rochester New York USA; ^25^ Department of Urology Mayo Clinic College of Medicine and Science Rochester New York USA; ^26^ Mayo Clinic College of Medicine and Science Mayo Clinic Cancer Center Rochester New York USA; ^27^ Department of Urology Zhongda Hospital Southeast University Nanjing China; ^28^ Department of Medicine Beth Israel Deaconess Medical Center and Harvard Medical School Boston Massachusetts USA; ^29^ Bioinformatics Platform, Department of Experimental Research, State Key Laboratory of Oncology in South China, Guangdong Key Laboratory of Nasopharyngeal Carcinoma Diagnosis and Therapy, Guangdong Provincial Clinical Research Center for Cancer Sun Yat‐sen University Cancer Center Guangzhou China; ^30^ Department of Pulmonary and Critical Care Medicine, Zhujiang Hospital Southern Medical University Guangzhou China; ^31^ Department of Endocrinology Singapore General Hospital Singapore Singapore; ^32^ Data Science and Artificial Intelligence Laboratory Singapore General Hospital Singapore Singapore; ^33^ Reconstructive Surgery and Regenerative Medicine Research Centre, Institute of Life Sciences Swansea University Medical School Swansea UK; ^34^ Welsh Centre for Burns and Plastic Surgery Morriston Hospital Swansea UK; ^35^ Department of Neurosurgery, Xiangya Hospital Central South University Changsha China; ^36^ National Clinical Research Center for Geriatric Disorders, Xiangya Hospital Central South University Changsha China; ^37^ Cancer Centre and Institute of Translational Medicine, Faculty of Health Sciences University of Macau Macau China; ^38^ MoE Frontiers Science Center for Precision Oncology University of Macau Macau China; ^39^ Department of Infectious Disease and Microbiology The University of Hong Kong‐Shenzhen Hospital Shenzhen China; ^40^ Department of Microbiology, State Key Laboratory of Emerging Infectious Diseases, Carol Yu Centre for Infection, School of Clinical Medicine, Li Ka Shing Faculty of Medicine The University of Hong Kong Hong Kong China

**Keywords:** generative AI, medical and life science contexts, reliability, standardized assessment guidelines

## Abstract

Generative artificial intelligence (AI) holds immense potential for medical applications, but the lack of a comprehensive evaluation framework and methodological deficiencies in existing studies hinder its effective implementation. Standardized assessment guidelines are crucial for ensuring reliable and consistent evaluation of generative AI in healthcare. Our objective is to develop robust, standardized guidelines tailored for evaluating generative AI performance in medical contexts. Through a rigorous literature review utilizing the Web of Sciences, Cochrane Library, PubMed, and Google Scholar, we focused on research testing generative AI capabilities in medicine. Our multidisciplinary team of experts conducted discussion sessions to develop a comprehensive 32‐item checklist. This checklist encompasses critical evaluation aspects of generative AI in medical applications, addressing key dimensions such as question collection, querying methodologies, and assessment techniques. The checklist and its broader assessment framework provide a holistic evaluation of AI systems, delineating a clear pathway from question gathering to result assessment. It guides researchers through potential challenges and pitfalls, enhancing research quality and reporting and aiding the evolution of generative AI in medicine and life sciences. Our framework furnishes a standardized, systematic approach for testing generative AI's applicability in medicine. For a concise checklist, please refer to Table S or visit GenAIMed.org.

## Highlights

- This work formulates the standardized testing and assessment guidelines for evaluating generative artificial intelligence (AI) reliability (STAGER) checklist, a 32‐item framework offering standardized assessment guidelines tailored for evaluating generative AI systems in medical and life science contexts.
- It consists of key aspects, including question collection, querying approaches, and assessment techniques.
- It enhances research quality and facilitates advances in this emerging field.

## Graphical abstract

This work formulates the standardized testing and assessment guidelines for evaluating generative artificial intelligence (AI) reliability (STAGER) checklist, a 32‐item framework offering standardized assessment guidelines tailored for evaluating generative AI systems in medical and life science contexts across key aspects including question collection, querying approaches, and assessment techniques. It enhances research quality and facilitates advances in this emerging field.

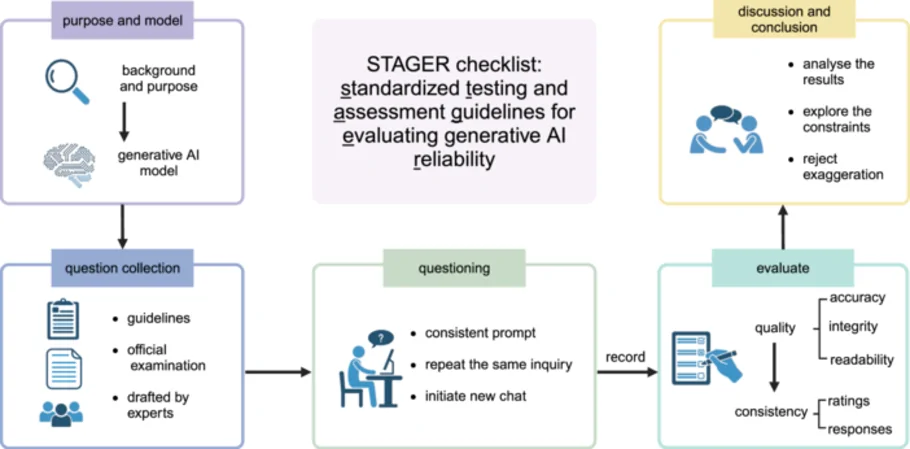

## 1 INTRODUCTION

**Figure 1:**
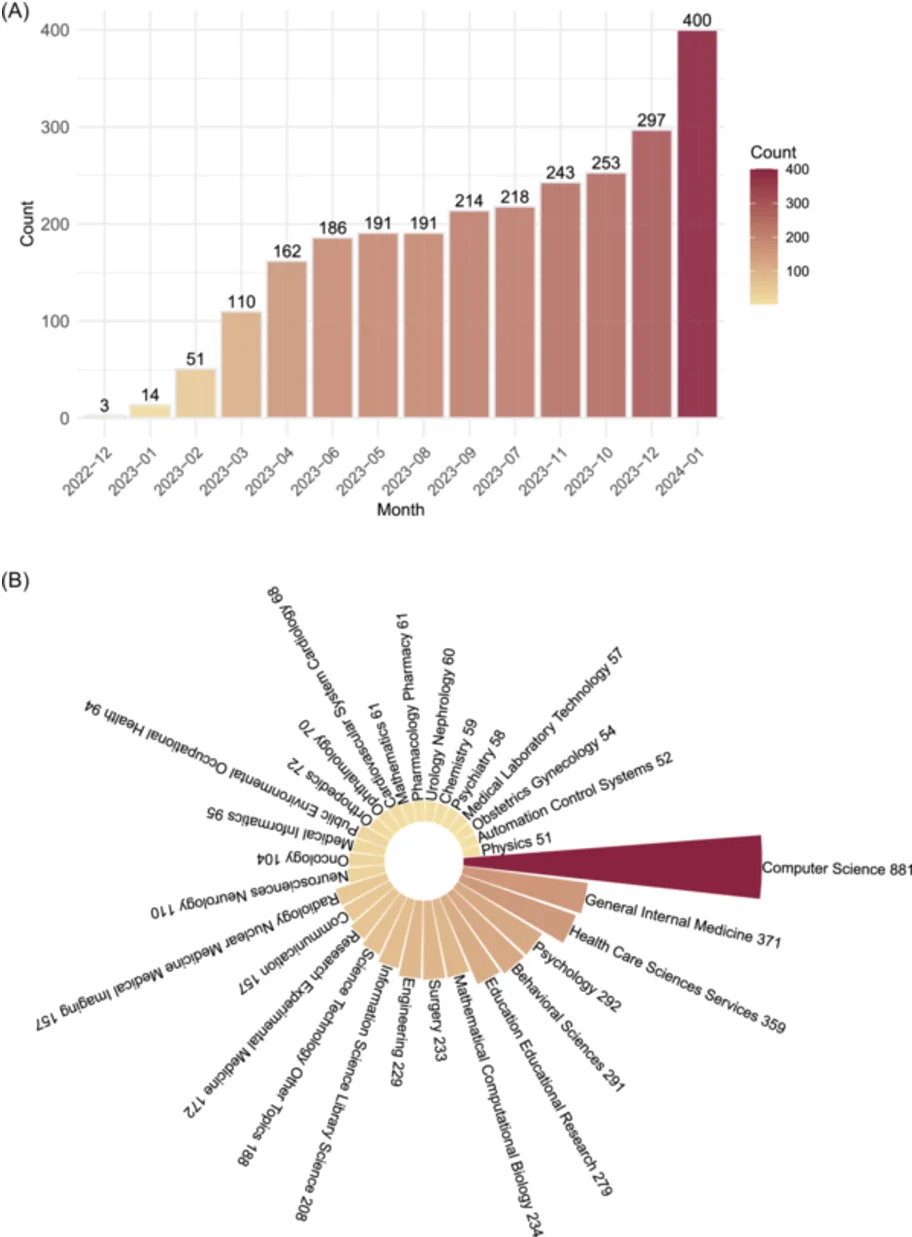
Publication records of a PubMed search using “ChatGPT” as the keyword. (A) It reveals a rapidly growing number of publications focused on generative AI, indicating a significant surge in interest and research in this field. (B) The top 20 areas involving the highest number of ChatGPT studies. ChatGPT, Chat generative pretrained transformer.

Generative artificial intelligence (AI), an increasingly prominent subfield of AI [1], boasts the remarkable ability to generate data across diverse formats, including text, images, audio, video, and code [2]. This versatility extends to its real‐time adaptability to novel task requirements through straightforward textual prompts [3, 4]. Taking a search of Chat generative pretrained transformer (ChatGPT) in PubMed as an example, the number of related studies shows a growing trend (Figure 1A). In the realm of medicine, generative AI stands out for its proficiency in rapidly processing multimodal information, such as medical texts and images, and the top 15 fields with the highest number of studies involving ChatGPT have a large proportion of medical‐related fields (Figure 1B). Generative AI can deliver responses to medical inquiries in natural language, offering critical support to medical professionals in diagnostic decision‐making and scientific research. Large‐language models such as ChatGPT [5], Google Bard [6], and New Bing [7] are perhaps the best‐explored generative AI tools in the medical field today.

Current research on the application of generative AI in the medical field encompasses a broad spectrum, ranging from assessing its grasp of medical knowledge and ability to pass medical examinations [8, 9] to aiding in providing initial medical counseling [10, 11] and swiftly providing pertinent medical information and recommendations [12, 13]. These studies underscore the vast potential for generative AI's deployment in healthcare [14, 15]. Nonetheless, a notable concern is that some published studies might exhibit methodological shortcomings and limitations in their assessment approaches. This can introduce varying degrees of bias into their findings. For instance, Fijačko et al. investigated ChatGPT's performance on the American Heart Association (AHA), Basic Life Support (BLS), and Advanced Cardiovascular Life Support (ACLS) exams and highlighted that ChatGPT was unable to pass these tests [16]. This conclusion, however, overlooked the fact that generative AI models often yield different responses to identical queries. A revised approach involving the repetition of the same question revealed that ChatGPT could indeed pass both exams with notable success [17]. Another critical gap lies in the lack of established frameworks for the systematic evaluation of generative AI in its capacity to address and apply solutions to medically relevant problems. There are widely adopted reporting guidelines for the evaluation of clinical trials involving AI intervention—such as the Consolidated Standards of Reporting Trials‐AI (CONSORT‐AI) [18] and the Standard Protocol Items: Recommendations for Interventional Trials‐AI (SPIRIT‐AI) [19] guidelines. There are also some new medical multimodal large language models for a specific medical field, such as a medical multimodal large language model for future pandemics [4]. However, generative AI interventions can deliver output that is simultaneously more nuanced and complex than general AI interventions. Early trials utilizing generative AI have typically focused on narrowly defined question‐answering use cases. However, with the rapid development of other generative AI tools, such as the vision‐language model, the potential use cases for generative AI will likely increase exponentially in the years to come. Therefore, there is still a role for a comprehensive and specific reporting guideline for generative AI interventions. Such a framework would not only standardize assessments but also significantly advance research in the realm of generative AI applications in medicine.

We propose a standardized methodological framework for reporting the output of generative AI systems in medical‐related fields. This framework serves as a comprehensive guide for the assessment of generative AI technologies, including gathering questions, framing them appropriately, conducting thorough outcome assessments, and so on. Recognizing the variation in generative AI's performance between multiple‐choice and open‐ended questions, our guide thoughtfully differentiates the approaches for handling these two question types. This distinction ensures a more nuanced and effective evaluation process. Covering critical aspects of the research process, our guidelines aim to assist researchers, medical professionals, and technology developers in conducting a thorough and precise evaluation of generative AI's capabilities in medical aptitude assessments, which includes scrutinizing aspects such as accuracy, integrity, and readability.

## 2 RESULTS

**Figure 2:**
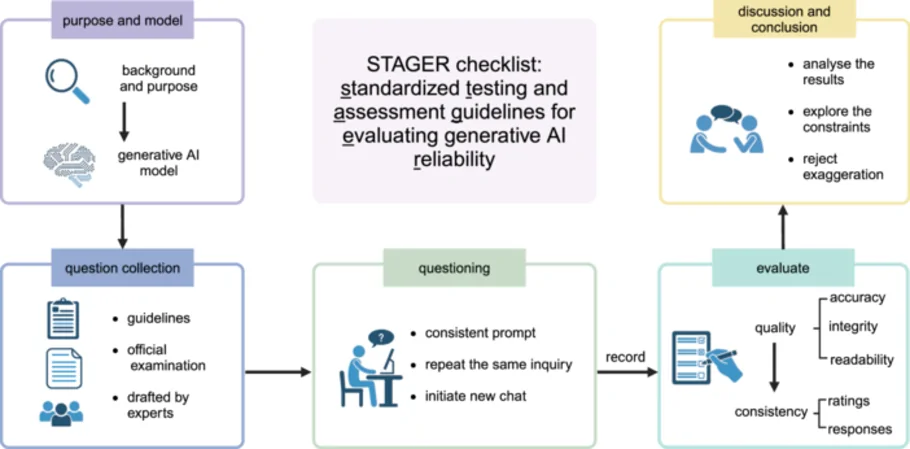
Schematic diagram outlining key components of the STAGER checklist for evaluating generative AI systems. This work formulates the STAGER checklist, a 32‐item framework offering standardized assessment guidelines tailored for evaluating generative AI systems in medical and life science contexts across key aspects, including question collection, querying approaches, and assessment techniques. It enhances research quality and facilitates advances in this emerging field. AI, artificial intelligence; STAGER, standardized testing and assessment guidelines for evaluating generative AI reliability.

We have developed a meticulously crafted checklist comprised of 32 distinct items, as depicted in Table 1. This checklist represents an extensive and intricate framework specifically designed for assessing the proficiency of generative AI within the medical field (Figure 2). Our framework thoroughly encompasses a variety of critical dimensions, such as the method of question collection, advanced questioning techniques, and a diverse range of assessment methods. These methods not only evaluate the accuracy and integrity of AI systems but also assess their ability to present information clearly and understandably.

**Table 1:** Evaluations and explanations of generative AI for medical applications.

Section/Topic	Item no	Recommendation	Explanations
Title	1	Identify the report as an article related to the research that evaluates generative AI's applicability in medicine.	Provide the reader with an initial understanding of the nature of the text.
Abstract	2	State the purpose of the research, the generative AI model used and its version, the source of the questions, methods, results, and conclusions.	Lay the foundation for readers to quickly understand the study and facilitate other researchers to critically analyze the design and results of this research.
Introduction			
Justification	3	Review existing relevant information and explain the background of the study.	Enable readers to grasp the central theme of the article.
Objectives	4	State‐specific objectives including the generative AI model used and its version, the training set used for generative AI, the source of the questions, the nature of research, and the limitations.	Provide the necessary framework for readers to understand the article.
Methods			
Question collection	5	Select the professional questions from guidelines, official examination question banks, and high‐frequency issues found via search engines like Google, or drafted by experts, ensuring that the questions cover specific subfields of medicine.	For guidelines or question banks, questions can be either manually selected or extracted using software, while using an API to select questions can reduce subjective errors and make more sense for the entire data set. When selecting questions from search engines, researchers may opt for frequently occurring ones. If the questions are drafted by experts, the experts need to have authority and experience in the relevant field.
	6	Ensure the questions are representative in terms of difficulty, type, and professionalism.	Enhance the universality of the study.
	7	Describe how the questions were collected, the number of questions, whether the questions were pre‐screened, the conditions of the screening, the modality of the input as well as the relevant format.	Input modes such as text, image, sound, video input, and so on, and related attributes (e.g., image resolution).
Agent	8	Record the model used, the version of the generative AI, and customized parameters such as temperature parameters, if applicable. State the strengths and weaknesses of the current version used and the rationale for assessing it.	The model used, and the version of the generative AI may have a significant influence on the result. Temperature is a parameter influencing text generation randomness. Higher temperatures yield more diverse and novel outputs, with increased unpredictability and potential inaccuracies. Lower temperatures produce consistent, predictable text closely aligned with training data but might lack creativity.
	9	If intend to report them as a functional series, it is recommended to report the relationship between model versions (e.g., whether it is a simple upgrade; if not, it is recommended to report the horizontal comparison results).	Reporting the relationship between model versions clarifies the evolution of the technology, helping users understand improvements or changes. Additionally, providing horizontal comparison results aids in comprehending the distinct capabilities and applications of each version.
Questioning	10	Use a consistent prompt with identically formatted patterns and provide the full prompt in the article.	Reduce the objective differences introduced by different questioning methods and the impact of such differences on the quality of answers. Providing the full prompt in the article ensures that the study is transparent and reproducible.
	11	Ask the same question multiple times and record each response.	Generative AI, known for delivering varied responses to identical queries, necessitates repeated questioning to gauge its consistency.
	12	Indicate whether the question is open‐ended or multiple‐choice.	Subjective and objective questions are assessed differently.
	13	Initiate a new chat for each question.	Prevent generative AI from being affected by context.
	14	Record the data the responses were collected.	Reduce the impact of performance differences between AI versions and the timing of knowledge updates.
Accuracy	15	Describe any methods employed for scoring accuracy when dealing with subjective questions.	Accuracy refers to the degree to which the response reflects or corresponds to reality or truth.
	16	Compare with reference answers, record the number of correct responses to each question, and calculate the rate of correct answers if you asked objective questions.	The more times the generative AI model responds correctly, the more robust it is considered to be.
Integrity	17	Describe any methods used to assess the integrity between responses.	Integrity refers to whether the response is comprehensive, detailed, and covers relevant information.
Readability	18	Describe any methods used to assess the readability of responses.	Reflect on the ease with which a text can be read and understood (e.g., clarity of language, the organization of structure, and grammatical and spelling accuracy).
Reviewers	19	Clarify the composition of reviewers and the rationale for this composition, which is recommended to be more than two experts from varied fields like medicine, AI, and interdisciplinary areas, along with stakeholders from ethics, sociology, and user groups.	Ensure the fairness and effectiveness of the evaluation process.
	20	Pay special attention to assessing the impenetrability of responses.	
	21	Evaluate the consistency across responses to the same question to assess whether the generative AI can steadily provide consistent responses.	
	22	Assess the consistency and reliability of reviewer ratings, avoiding significant differences in the subjective scores among reviewers.	A way to effectively monitor model performance, helping detect if the model has erratic behavior.
Results			
Results selection	23	Describe the results of the search process, from the number of questions collected to the final results, ideally using a flow diagram.	Uncovering its performance in specific subdomains is critical to a deeper understanding of the value and limitations of AI applications in medicine.
Study characteristics	24	State all studies included in the analysis and detail their characteristics.	
Results of individual studies	25	Present results for accuracy, completeness, and readability for each study, recommending the use of tables or charts for presentation.	
Results of syntheses	26	Present results of all statistical syntheses conducted and results of analyses conducted to explore possible causes of heterogeneity among study results.	
Discussion			
Interpretation	27	Analyze the results according to the study objectives.	Comprehensively analyze the performance of generative AI in terms of accuracy, completeness, and readability.
Strengths and limitations	28	Describe the advantages of the research.	To make the reader understand the importance of the study.
	29	Explore constraints of the research, acknowledging possible origins of partiality or inaccuracy.	Enhance the understanding of the scope, accuracy, and applicability of the research findings.
	30	Engage in rational discussion and reject exaggeration.	An honest and rational expression is necessary to maintain academic norms and advance knowledge.
Conclusion	31	Provide a condensed conclusion that summarizes the study's main findings, reiterates its importance, and indicates directions or recommendations for future research.	Provide direction for future research and help promote the further development and application of generative AI in the medical field.
Other information			
Funding and sponsorship	32	Provide the origin of financial support and the function of the sponsors for the current investigation, as well as for the initial research if relevant to the foundation of this article.	Maintain the objectivity and transparency of the research.

Abbreviations: AI, artificial intelligence; API, application programming interface.

The comprehensive nature of this checklist ensures a detailed and in‐depth evaluation of generative AI's capabilities in managing and interpreting medical data and scenarios. By exploring these diverse dimensions, we have gained valuable insights into the strengths and potential limitations of AI when it comes to processing and interpreting complex medical information. This understanding is crucial in optimizing AI applications in healthcare settings.

Furthermore, we provide an exhaustive explanation for each item listed in the checklist, also detailed in Table 1. These explanations are crafted to elucidate the underlying rationale and importance of each step in the evaluation process. We aim to offer clear guidance to researchers, aiding them in effectively navigating the multifaceted challenges they may encounter in their investigative pursuits in the realm of medical AI. This guidance is instrumental in ensuring that researchers can conduct thorough and meaningful evaluations, ultimately contributing to the enhancement and reliability of generative AI applications in medical research and practice.

## 3 DISCUSSION

In crafting these guidelines, we have centered our efforts on developing a comprehensive 32‐item checklist meticulously tailored to assess the applicability of generative AI in medicine and life sciences. This guide's innovation is manifest in its broad assessment dimensions, which encompass crucial aspects such as question collection, questioning approaches, and diverse assessment methods. This holistic approach facilitates a deeper understanding and assessment of generative AI's performance in medical contexts, thereby propelling advancements in the field.

The checklist is conceived with an acute awareness of the current challenges in applying generative AI within the medical field. One significant challenge is the opacity in generative AI's data processing and information generation. This lack of transparency often leads to difficulties in deciphering and interpreting the outcomes, potentially undermining the credibility and usability of AI in medical applications. Our checklist addresses this by providing a standardized framework that rigorously assesses these critical aspects, thereby enhancing the quality and reliability of the research. Another major challenge is the variability in the processes of question collection, framing, and conducting comprehensive outcome assessments. Such variations, stemming from the diverse methodologies employed by different researchers, introduce a level of subjectivity that could skew assessment results. Our checklist confronts this issue by offering a detailed and practical framework. It emphasizes crucial factors like the collection of questions, the choice of AI agents, and the approaches to posing questions, all of which significantly influence the outcomes. This detailed approach effectively counters concerns of subjectivity, ensuring more objective and reliable assessments.

Also, we offer the following recommendations for AI model developers: While emphasizing data quality and diversity, we encourage developers to explore innovative methodologies that may deviate from traditional models. This flexibility can lead to breakthroughs in AI applications. In addition, the integration of generative AI in medical ethics, while promising, requires careful consideration of their epistemic limitations [20, 21]. Consequently, we recommend a flexible approach to ethical considerations, adapting to diverse contexts while firmly adhering to core principles like patient privacy and data security. This balance is key in a field where ethical challenges evolve as rapidly as technology.

Moreover, our checklist is designed to maintain a balance between methodological rigor and flexibility, crucial for the rapidly evolving field of AI in medicine. We have not provided a scoring rule, aiming to fuel the creativity of researchers. For example, one study explored the potential of large language models as tools against medical disinformation [22]. This approach encourages innovative applications and interpretations of AI technology, allowing for groundbreaking developments that might not be fully captured by a rigid scoring system. By embracing this open‐ended approach, our framework fosters an environment where unconventional ideas can be tested and refined, thereby accelerating the advancement of AI in healthcare. This flexibility also permits the integration of new techniques and findings, ensuring that our guidelines remain relevant and effective as the field continues to grow and change.

Furthermore, the detailed explanation of each checklist item in our guidelines is not just about aiding comprehension. It also plays a vital role in minimizing subjective interpretation variances and bolstering the reproducibility of assessments. This guidance empowers researchers to identify and address potential challenges in their studies, which is instrumental in elevating the quality of research. By providing this comprehensive framework and guidance, we aim to pave the way for more nuanced, effective, and reliable use of generative AI in medical research. This approach is vital in ensuring that AI technologies not only advance in capability but also align with the stringent requirements and ethical considerations intrinsic to medical science.

## 4 CONCLUSION

The assessment framework delineated in these guidelines introduces a standardized and systematic method for evaluating generative AI research in medical applications, with an emphasis on elevating the quality of research reports. This framework is pivotal in nurturing the development of generative AI within medical contexts, ensuring that AI systems are not only innovative but also valid and reliable for practical use. By providing a clear set of criteria for evaluation, it addresses the need for transparency and rigor in AI research, which is crucial in a field where accuracy and dependability are paramount. Furthermore, this framework is expected to foster academic collaboration and intellectual exchange, creating a fertile ground for cross‐disciplinary partnerships. Such collaboration is essential for the continued evolution of generative AI technology in medical applications, ensuring that it remains cutting‐edge, relevant, and aligned with the ever‐changing landscape of medical science. Through this framework, we aim to contribute significantly to the sustained advancement of AI technology in medical applications, enhancing its role in revolutionizing medical research and practice.

## 5 METHODS

### Review of related literature

To craft a detailed and formidable framework for assessing the efficiency of generative AI in medical competency testing, we embarked on an exhaustive research endeavor. We delved into several renowned databases, including the Web of Sciences, Cochrane Library, PubMed, and Google Scholar, conducting a thorough investigation for pertinent studies within the realm of generative AI's application in medical settings. Our objective was to amass a diverse array of perspectives and methodologies prevalent in contemporary research. This strategic approach was pivotal in guaranteeing the pertinence and thoroughness of our proposed checklist.

### Criteria for study selection

The extraction process from these databases was guided by stringent criteria, focusing on studies that demonstrated significant insights into the assessment and application of generative AI in medical competency testing. Our search parameters included a range of keywords and phrases specifically tailored to capture the most relevant and current research in this rapidly evolving field. This extensive literature review not only provided us with a plethora of potential checklist items but also offered a deep understanding of the existing challenges and gaps in the evaluation of generative AI within medical applications.

### Formation of interdisciplinary expert team

Following the literature review, we assembled an interdisciplinary team of experts, each bringing a unique and critical perspective to the table. This team comprised specialists in life sciences, clinical medicine, and medical engineering, all of whom were active users of generative AI technologies. Their diverse backgrounds and practical experience in using AI tools in medical settings were instrumental in providing a well‐rounded approach to checklist development.

### Discussion of the items in the checklist

The team engaged in a series of structured and in‐depth discussions, following the protocols outlined in the “Guidance for Developers of Health Research Reporting Guidelines.” These sessions were not only aimed at validating and refining the initially extracted checklist items but also at integrating the varied insights and experiences of our team members. Each session was meticulously planned to ensure a focused and productive discussion, with specific agenda items and checklist components assigned for review and debate.

### Review and refinement of items

During these discussions, the team rigorously reviewed each potential checklist item, considering its relevance, applicability, and importance in evaluating generative AI's proficiency in medical competency testing. Emphasis was placed on ensuring that each checklist item was clear, measurable, and aligned with the highest standards of medical research and AI application. The team also focused on the potential for each item to address the specific challenges and nuances of generative AI in a medical context. This involved a critical analysis of each item's ability to assess not only the technical proficiency of AI systems but also their practical utility, ethical considerations, and impact on clinical outcomes.

### Iterative development and collaborative wisdom

The collaborative process was iterative, with each session building upon the insights and feedback from previous discussions. This iterative approach allowed for continuous refinement of the checklist, ensuring that each item was not only individually robust but also coherent within the overall framework. The team's collective expertise and the dynamic nature of the discussions were instrumental in developing a comprehensive and actionable set of guidelines. This collaborative process ensured that the final checklist was a product of collective wisdom, balancing theoretical underpinnings with practical insights and clinical relevance.

Through this rigorous and collaborative methodology and following Appraisal of Guidelines for Research and Evaluation (AGREE) [23], we developed a comprehensive set of guidelines for evaluating generative AI's proficiency in medical competency testing. This framework not only meets the current needs of the field but is also adaptable to future advancements and challenges in the application of AI in medicine.

## AUTHOR CONTRIBUTIONS

**Jinghong Chen**: Visualization; writing—original draft; conceptualization; methodology; investigation; validation. **Lingxuan Zhu**: Conceptualization; writing—original draft; writing—review and editing; methodology; validation; investigation. **Weiming Mou**: Conceptualization; methodology; writing—original draft; writing—review and editing; investigation; validation. **Anqi Lin**: Writing—original draft; writing—review and editing; validation; conceptualization; methodology. **Dongqiang Zeng**: Writing—review and editing. **Chang Qi**: Writing—review and editing. **Zaoqu Liu**: Writing—review and editing. **Aimin Jiang**: Writing—review and editing. **Bufu Tang**: Writing—review and editing. **Wenjie Shi**: Writing—review and editing. **Ulf D. Kahlert**: Writing—review and editing. **Jianguo Zhou**: Writing—review and editing. **Shipeng Guo**: Writing—review and editing. **Xiaofan Lu**: Writing—review and editing. **Xu Sun**: Writing—review and editing. **Trunghieu Ngo**: Writing—review and editing. **Zhongji Pu**: Writing—review and editing. **Baolei Jia**: Writing—review and editing. **Che Ok Jeon**: Writing—review and editing. **Yongbin He**: Writing—review and editing. **Haiyang Wu**: Writing—review and editing. **Shuqin Gu**: Writing—review and editing. **Wisit Cheungpasitporn**: Writing—review and editing. **Haojie Huang**: Writing—review and editing. **Weipu Mao**: Writing—review and editing. **Shixiang Wang**: Writing—review and editing. **Xin Chen**: Writing—review and editing. **Loïc Cabannes**: Writing—review and editing. **Gerald Sng Gui Ren**: Writing—review and editing. **Iain S. Whitaker**: Writing—review and editing. **Stephen Ali**: Writing—review and editing. **Quan Cheng**: Writing—review and editing; validation. **Kai Miao**: Validation; writing—review and editing. **Shuofeng Yuan**: Validation; writing—review and editing. **Peng Luo**: Validation; writing—review and editing.

## CONFLICT OF INTEREST STATEMENT

The authors declare no conflict of interest.

## ETHICS STATEMENT

No animals or humans were involved in this study.

## Supporting information


**Table S1:** Evaluations of generative AI for medical applications.

## Data Availability

Data sharing is not applicable to this article as no new data were created or analyzed in this study. No new data or scripts were used in this paper. Supplementary information (tables, graphical abstract, slides, videos, Chinese translated version, and update materials) is available online DOI or http://www.imeta.science/imetaomics/.
